# Changes in the *Ixodes ricinus* microbiome associated with artificial tick feeding

**DOI:** 10.3389/fmicb.2022.1050063

**Published:** 2023-01-10

**Authors:** Nina Militzer, Sophia Pinecki Socias, Ard M. Nijhof

**Affiliations:** ^1^Institute for Parasitology and Tropical Veterinary Medicine, Freie Universität Berlin, Berlin, Germany; ^2^Veterinary Centre for Resistance Research, Freie Universität Berlin, Berlin, Germany

**Keywords:** *Ixodes* *ricinus*, *Midichloria*, *Rickettsia helvetica*, *Spiroplasma*, *in vitro* feeding, artificial feeding, vitamin B

## Abstract

Artificial tick feeding systems (ATFS) can be used to study tick biology and tick-pathogen interactions. Due to the long feeding duration of hard ticks, antibiotics are commonly added to the *in vitro* blood meal to prevent the blood from decaying. This may affect the ticks’ microbiome, including mutualistic bacteria that play an important role in tick biology. This effect was examined by the consecutive feeding of *Ixodes ricinus* larvae, nymphs, and adults *in vitro* with and without the supplementation of gentamicin and in parallel on calves. DNA extracted from unfed females was analyzed by 16S rRNA sequencing. The abundance of *Candidatus Midichloria mitochondrii*, *Rickettsia helvetica* and *Spiroplasma* spp. was measured by qPCR in unfed larvae, nymphs, and adults. Larvae and nymphs fed on calves performed significantly better compared to both *in vitro* groups. Adults fed on blood supplemented with gentamicin and B vitamins had a higher detachment proportion and weight compared to the group fed with B vitamins but without gentamicin. The detachment proportion and weights of females did not differ significantly between ticks fed on calves and *in vitro* with gentamicin, but the fecundity was significantly higher in ticks fed on calves. 16S rRNA sequencing showed a higher microbiome species richness in ticks fed on calves compared to ticks fed *in vitro*. A shift in microbiome composition, with *Ca*. *Midichloria mitochondrii* as dominant species in females fed as juveniles on calves and *R*. *helvetica* as the most abundant species in females previously fed *in vitro* was observed. Females fed *in vitro* without gentamicin showed significant lower loads of *Ca*. M. mitochondrii compared to females fed *in vitro* with gentamicin and ticks fed on calves. *Spiroplasma* spp. were exclusively detected in female ticks fed on cattle by qPCR, but 16S rRNA sequencing results also showed a low abundance in *in vitro* females exposed to gentamicin. In conclusion, the employed feeding method and gentamicin supplementation affected the ticks’ microbiome composition and fecundity. Since these changes may have an impact on tick biology and vector competence, they should be taken into account in studies employing ATFS.

## 1. Introduction

Artificial tick feeding systems (ATFS) in which ticks are fed on artificial membranes or animal skin *in vitro* have been widely used, for instance in studies on tick biology, tick-pathogen interactions and the screening of anti-tick compounds under controlled laboratory conditions (Waladde et al., 1979; Kuhnert et al., 1995; Krober and Guerin, 2007; Antunes et al., 2014; Król et al., 2021; Militzer et al., 2021). In addition, ATFS contribute to the 3R principle to Reduce, Refine and Replace animal experimentation in science. Membrane-based ATFS typically consist of a tick containment unit, a membrane which mimics the skin, a blood meal and a heating device to warm the blood to 37–39°C (Nijhof and Tyson, 2018).

Hard ticks have a long feeding duration of several days to weeks and this period is usually extended when ticks are fed *in vitro* (Kuhnert et al., 1995; Militzer et al., 2021). This makes the use of ATFS challenging as the blood meal may decay due to microbial growth. The blood meal is therefore regularly changed and routinely treated with antimicrobials such as penicillin, streptomycin, rifampicin, phosphomicin, ciprofloxacin and gentamicin, or antimycotic substances such as amphotericin b or nystatin to prevent the blood from decaying (Kemp et al., 1975; Kuhnert et al., 1995; Krober and Guerin, 2007; Oliver et al., 2021). It is plausible that the addition of antimicrobial compounds to the blood meal will also affect the tick microbiome composition, but little is known about the extent of this effect.

It was previously shown that a dysbiosis of the ixodid tick microbiome after injection of ticks with antibiotics resulted in reduced fecundity, feeding, survival and/or development (Zhong et al., 2007; Kurlovs et al., 2014; Guizzo et al., 2017; Li et al., 2018; Ben-Yosef et al., 2020; Zhong et al., 2021). Alternative antibiotic treatment methods, such as feeding ticks on antibiotic-treated animals were also reported to have a negative effect on tick fecundity (Clayton et al., 2015; Zhang et al., 2017; Duron et al., 2018). Dysbiosis could also be induced by wash procedures or sterile maintenance (Narasimhan et al., 2014; Hamilton et al., 2021; Hurry et al., 2021). To our knowledge, only a single study has so far described the dysbiosis of hard ticks by feeding *Ixodes scapularis* female ticks on blood treated with antibiotics through an artificial membrane (Oliver et al., 2021). Changes to the tick microbiome were also shown to affect the vector competence of ticks: dysbiosed *I*. *scapularis* larvae were for instance less prone to *Borrelia* colonization (Narasimhan et al., 2014), whereas *A*. *phagocytophilum* colonization was increased in *I*. *scapularis* nymphs fed on gentamicin-treated mice infected with *A*. *phagocytophilum* (Abraham et al., 2017).

In Europe, *Ixodes ricinus* is the most widely distributed tick in Europe and the main vector for tick-borne pathogens causing Lyme Borreliosis and Tick-Borne Encephalitis in humans. Like all hard ticks, *I*. *ricinus* requires a blood meal in each parasitic life stage, i.e., as larvae, nymph and adult, in order to develop and reproduce. In *I*. *ricinus*, *Candidatus* M. mitochondrii (hereafter *M*. *mitochondrii*) is the most commonly reported maternally inherited symbiont (Gofton et al., 2015; Aivelo et al., 2019). Other bacteria associated with *I*. *ricinus* are *Rickettsiella* spp., *Borrelia* spp., *Spiroplasma*, *Rickettsia* spp., *A*. *phagocytophilum*, and *Candidatus* Neoehrlichia (van Overbeek et al., 2008; Gofton et al., 2015; Aivelo et al., 2019; Garcia-Vozmediano et al., 2021; Lejal et al., 2021). Maternally inherited bacterial endosymbionts play an important role in nutrition, defense and immune pathways. As the blood meal is lacking B vitamins and co-enzymes, it has been suggested that these and other nutrients could be provided to the tick by their endosymbionts (Gottlieb et al., 2015; Smith et al., 2015; Duron et al., 2018; Duron and Gottlieb, 2020). Biosynthesis pathways for certain B vitamins and cofactors were shown to be present in the genomes of some endosymbionts, including *M*. *mitochondrii* (Duron et al., 2017; Olivieri et al., 2019; Buysse et al., 2021).

The objective of this study was to compare the *in vitro* feeding of *I*. *ricinus* on bovine blood using an ATFS to a control group of *I*. *ricinus* ticks fed on cattle (C). To assess the influence of antibiotic treatment on tick feeding parameters in the ATFS, we compared ticks fed *in vitro* on gentamicin-treated blood (IVG^+^) to ticks fed *in vitro* on blood without antibiotics (IVG^−^). 16S rRNA sequencing was used to identify microbial communities in the ticks. The abundance of three of the most common species present in unfed females: *Rickettsia helvetica*, *M*. *mitochondrii* and *Spiroplasma* spp. was subsequently quantified by qPCR for samples from unfed larvae, nymphs, females and males.

## 2. Materials and methods

### 2.1. Ticks and the *in vivo* feeding

All *I*. *ricinus* ticks originated from a laboratory colony of the Institute for Parasitology and Tropical Veterinary Medicine of the Freie Universität Berlin. The feeding of each life stage was done in parallel for the IVG^−^, IVG^+^ and C groups. The study started with the feeding of *I*. *ricinus* F_0_-larvae at 2–5 months post hatching in May–June 2019. The F_0_-larvae were the offspring of four females from the laboratory colony. Nymphs that molted from these larvae were fed at 3–4 months post molting between October and November 2019, while the resulting adults were fed between 2 and 8 months after molting between July and September 2020. Ticks of the C group were fed on the ears of tick-naïve Holstein-Friesian calves that were 3.5–4.5 months of age. The estimated number of unfed larvae used was calculated by dividing the weight of the larvae batch by the calculated mean weight of a single unfed larva measured by an analytic scale. For the feeding, the base of each ear was covered with fabric-based tape (Leukoplast, BSN medical, Hamburg, Germany) after which linen ear bags containing equal amounts of ticks were placed over the ears. The ear bags were subsequently attached to the tape at the base of the ear by a second piece of Leukoplast. Detached ticks were collected twice daily. All animal experiments were approved by the regional authority for animal experimentation (LaGeSo, Berlin, 0387/17).

### 2.2. *In vitro* feeding (IVG^+^ and IVG^−^)

All feeding experiments were performed as previously reported (Militzer et al., 2021). Aseptically withdrawn heparinized bovine blood was supplemented with 2 g/L glucose and 0.1 M adenosine triphosphate (ATP, Carl Roth). Due to previous experiences with the artificial feeding of *I*. *ricinus* adults with blood supplemented with gentamicin, such as long feeding durations and the observation that some attached ticks turned black and died, we decided to supplement the blood meals for adults of both groups (IVG^+^ and IVG^−^) with B vitamin components (Militzer et al., 2021). For the ticks fed on blood supplemented with antibiotics (IVG^+^), 5 μg gentamicin (Cellupur, Carl Roth, Karlsruhe, Germany) was added per mL of blood.

After feeding to repletion, larvae and nymphs of all groups (IVG^+^, IVG^−^, and C) were stored at room temperature (RT) and > 90% relative humidity (RH) under a natural light–dark regime. Adult ticks were stored at 20°C, >90% RH in darkness.

### 2.3. Sample preparation and DNA extraction

Immediately before each feeding experiment, unfed tick samples were collected and stored at −20°C. Prior to DNA extraction, all unfed ticks were surface-sterilized as previously described (Binetruy et al., 2019b). This was performed by washing the ticks in 1% commercial bleach for 30 s, followed by a rinsing for 1 min in three successive baths of DNA-free water. Individual females were quadrisected and nymphs and males were bisected to facilitate subsequent homogenization by crushing with a pestle. Only sterile tubes, scalpel blades and pestles were used. Genomic DNA extraction was performed using the Nucleospin Tissue XS kit (Macherey-Nagel) following the manufacturer’s protocol, with an overnight lysis step and a final elution volume of 40 μL. All eluates were evaporated at RT for 10 min to remove residual ethanol. Extraction was performed for individual nymph and adult samples (*n* = 5–8 per experimental group) or in batches for larvae (*n* = 13–20). Negative controls were included for each batch of extracted DNA and consisted of tubes without tick material that were processed together with the tick samples.

### 2.4. Bacterial 16S rRNA sequencing

DNA from unfed ticks and negative controls were used for further NGS analysis. To amplify a 466 bp fragment spanning the V3-V4 region of bacterial 16S rRNA, primers 341F (5′-CCTAYGGGRBGCASCAG-3′) and 806R (5′-GGACTACNNGGGTATCTAAT-3′) were used. The whole amplification, library preparation and sequencing workflow including prior quality control was performed by Novogene Inc (Beijing, China). Briefly, quality control (QC) was performed on a 1% agarose gel electrophoresis. Tick DNA was subsequently diluted to 1 ng/μL using sterile water and subjected to PCR using Phusion High-Fidelity PCR Master Mix (New England Biolabs), followed by agarose gel electrophoresis. Only samples showing a bright band between 400 and 460 bp were used for library generation. The purification of PCR product mixtures was performed using the Qiagen Gel Extraction kit (Qiagen, Germany). Libraries were generated by NEBNext Ultra DNA Library Prep Kit for Illumina (New England Biolabs) and quantified by Qubit and quantitative PCR (qPCR). Sequencing was performed on a Novaseq 6000 (Illumina) with a sequencing depth of 50 k raw reads per sample.

### 2.5. Bioinformatics and statistical analyses of NGS data

Processing of the sequence data including Operational Taxonomic Unit clustering was performed by Novogene Inc. Paired-end reads were merged by FLASH software (V1.2.7.) (Magoc and Salzberg, 2011) and further quality-filtered by QIIME (V1.7.0.) (Caporaso et al., 2010). Chimera sequences were identified and eliminated using the UCHIME algorithm (Edgar et al., 2011). Sequence analysis was performed by Uparse software (V7.0.1001). Mothur software was used against SSUrRNA database of the SILVA reference database (Wang et al., 2007; Quast et al., 2013) for species annotation, with a cut-off at ≥97% similarity. MUSCLE (3.8.31) (Edgar, 2004) software was used for further phylogenetic analyses. Alpha and beta diversity were analyzed by R (V 4.2.2). For data processing, the phyloseq package was used (McMurdie and Holmes, 2013). We further used the decontam package to identify possible contaminants by comparing the OTU abundance of negative controls to samples (Davis et al., 2018). Here, the prevalence method with a threshold of 0.5 was used. This was followed by trimming OTUs which were not present in any samples from our data subset. Further analysis was performed with this data subset. Alpha diversity for species richness included Chao1 and abundance-based coverage estimator (ACE), while for species diversity Shannon and Simpson index, the index of sequencing depth and observed species were included. Here, the Wilcoxon test was performed for statistical analysis and the graphs were computed by phyloseq package, ggplot2 and ggpubr packages (Wickham, 2011; McMurdie and Holmes, 2013; Kassambara, 2020). Beta diversity measures included weighted and unweighted UniFrac, focusing on relative abundances by using the Bray-Curtis distance measure computed by the phyloseq package. Further, the Non-Metric Multidimensional Scaling (NMDS) was computed by phyloseq package and a Principal Component Analysis (PCA) was computed and visualized by the MicrobiotaProcess package (McMurdie and Holmes, 2013; Xu and Yu, 2022). Statistical analyses for dissimilarity measures were performed by Permutational Multivariate Analysis of Variance (PERMANOVA) by the vegan package (*n* = 999 permutations; Oksanen et al., 2013). For beta diversity graphs, the phyloseq package was used (McMurdie and Holmes, 2013). For all statistical tests, a statistical significance level at *p* < 0.05 was set.

### 2.6. Sequencing

The *Rickettsia* and *Spiroplasma* spp. detected by bacterial 16S rRNA sequencing were further identified by amplifying a ~ 499 bp region of the *Rickettsia gltA* gene and a ~ 561 bp fragment of the *Spiroplasma* DNA gyrase subunit A (*gyrA*) gene (Table 1), followed by amplicon sequencing (LGC Genomics, Berlin, Germany) and BLASTn analysis.

**Table 1:** Primers used in this study.

**Organism**	**Target gene**	**Primer name**	**Sequence (5’–3’)**	**Annealing temperature for PCR (°C)**	**Product size (bp)**	**Reference**
*Ca*. Midichloria mitochondrii	*gyrB*	gyrB-F	CTTGAGAGCAGAACCACCTA	61.5	125	Sassera et al. (2008)
		gyrB-R	CAAGCTCTGCCGAAATATCTT			
*Ixodes ricinus*	*Calreticulin*	calF	ATCTCCAATTTCGGTCCGGT	64.5	109	
		calR	TGAAAGTTCCCTGCTCGCTT			
*Rickettsia* spp.	*gltA*	Rickettsia_gltA_F1	GCTCTTCTCATCCTATGGCTATTA	59.1	499	This study
		Rickettsia_gltA_R2	TCCTTAGCTTTAGCTATATATTTAGG			
*Rickettsia helvetica*	*gltA*	Rhelvetica_qPCR_F2	GGAAGCAGACTACAAACTTACTGC	–	173	This study
		Rhelvetica_qPCR_R2	CTTTATATTTCGTACAAGGCGTTG			
*Spiroplasma* spp.	*rpoB*	Spiro_rpoB_qPCR_F1	CCAAAAGGTCAAACACAATCAAC	62.1	127	This study
		Spiro_rpoB_qPCR_R1	TACCTTGAACAATTCCAGCACC			
*Spiroplasma*	*gyrA*	Spixo_gyrA_F2	CCAGATGCAAGAGATGGATTG	56	561	Binetruy et al. (2019a)

### 2.7. Plasmid DNA for standard curves

To generate plasmid DNA for standard curves that were used in the qPCR, PCR products were amplified using S7 Fusion polymerase (Mobidiag, Espoo, Finland). Each reaction mixture consisted of 5 μL of 5X HF buffer, 1 μL of each forward and reverse primer (10 μM), 0.5 μL of dNTP (2 mM), 0.25 μL polymerase, 1 μL DNA and nuclease-free water up to a reaction volume of 25 μL. Cycling conditions were 98°C for 8 s followed by 35 cycles of 94°C for 5 s, annealing for 20 s, and 72°C for 15 s, with a final extension step at 72°C for 1 min. Amplicons of the expected size were cleaned using the DNA Clean & Concentrator-5 kit (Zymo Research, Freiburg, Germany) and cloned in the pSC-B-amp/kan vector (Strataclone Blunt Cloning Kit, Agilent). Plasmids were isolated using the GenUp Plasmid Kit (Biotechrabbit, Berlin, Germany) and sequenced by LGC Genomics. Ten-fold serial dilution stocks with known copies of each plasmid DNA were prepared and stored at −20°C.

### 2.8. Quantification of endosymbionts

To measure the relative level of three of the most abundant bacteria identified by bacterial 16S rRNA sequencing in all unfed life stages (including the same female tick DNA samples as used for 16S sRNA sequencing), we performed qPCRs using the primers listed in Table 1. Novel primers were manually designed using NetPrimer software^1^ based on nucleotide sequence alignments made in BioEdit 7.0.5.3.^2^ qPCR reaction mixtures consisted of 10 μL Luna Universal mastermix (New England Biolabs, Frankfurt am Main, Germany), 1 μL of each primer (10 μM), 1 μL DNA and 7 μL nuclease-free water. Cycling conditions were 95°C for 10 min, followed by 50 cycles of 95°C for 15 s and annealing/elongation at 60°C for 1 min in a CFX96 cycler (Bio-Rad Laboratories GmbH, Feldkirchen, Germany). All samples were run in technical duplicates. A no-template control and serial dilutions of plasmid DNA were included in each run. Results were normalized against the *I*. *ricinus calreticulin* (*cal*) gene as a reference gene in CFX Maestro software (Bio-Rad).

### 2.9. Statistical analyses for feeding and quantitative PCR data

For tick feeding experiments, analyses were computed in R (V 4.2.2.) (R Core Team, 2013) either by the Mann–Whitney U test or the *t*-test with Welch-Correction depending on normal distribution, the Z-test for proportions followed by degrees of freedom (df) and Chi-Square (χ2). Confidence intervals (CI) were computed at the 95% level and CIs for proportions were computed by the binom.wilson function from the epitools package (V 0.5–10.1). Graphs of feeding parameters and relative bacterial abundance were produced using the ggplot2 package (3.3.1) with a significance level of *p* < 0.05 (Wickham, 2011). The graphs and data analysis concerning the qPCR data on target gene/ housekeeping gene ratio were performed in GraphPad Prism 9.3.1 (Graphpad Software Inc., La Jolla, United States), for which a Mann–Whitney test was performed and a value of *p* < 0.05 was considered as statistically significant.

## 3. Results

### 3.1. Artificial tick feeding with and without antibiotics and tick feeding on cattle

All consecutive life stages (larvae, nymphs, adults) were successfully fed in the IVG^+^ and the IVG^−^ group. For the C group, feeding of consecutive larvae and nymphs on calves was also successful but feeding of the adult ticks on a calf failed for unknown reasons. Adults originating from a different larval batch to obtain comparative data for the *in vitro* feeding were used instead. Since only the microbiome of unfed larvae, unfed nymphs and unfed adult ticks originating from the same larval batches were analyzed, this did not affect the results from 16S rRNA sequencing or the qPCR analysis.

Overall, we fed an estimated number of 1,505 (IVG^−^), 709 (IVG^+^), and 1160 (C) F_0_-larvae. For the consecutive feeding of nymphs that molted from these larvae, a total of 96 (IVG^−^), 136 (IVG^+^) and 200 (C) nymphs were used. These ticks were again fed after molting as adults. Here, 25 (IVG^−^), 10 (IVG^+^) and 100 (C) females were fed.

#### 3.1.1. Larvae feeding

**Figure 1:**
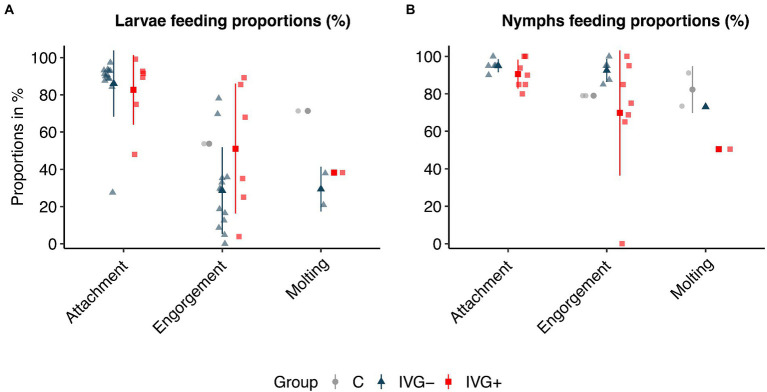
Attachment, engorgement, and molting proportions of *Ixodes ricinus* larvae **(A)** and nymphs **(B)**. Proportions are presented in % per feeding unit or per animal experiment. Bars present means ± standard deviation. IVG^+^, *in vitro* feeding with gentamicin; IVG^−^, *in vitro* feeding without gentamicin; C, control feeding *in vivo* on calves.

In general, we observed a statistically significant positive effect of gentamicin supplementation in the IVG^+^ larvae group compared to IVG^−^ for the proportion of engorged larvae and molting proportion per engorged larvae (Figure 1A; Supplementary Table S1). Although IVG^+^ larvae showed a statistically significant higher engorgement proportion than control ticks, the molting proportion was significantly higher for larvae fed on calves.

#### 3.1.2. Nymphal feeding

**Figure 2:**
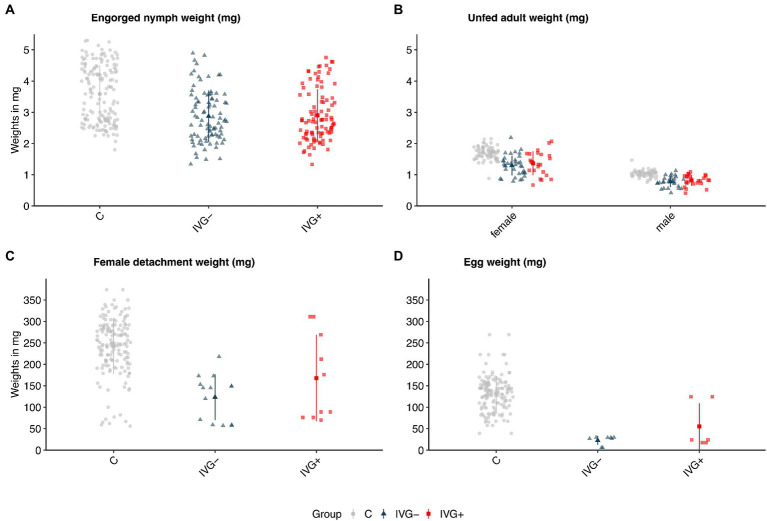
Weights (in mg) of **(A)** engorged *Ixodes ricinus* nymphs, **(B)** molted unfed adult *I*. *ricinus* females and males, **(C)** engorged and detached *I*. *ricinus* females, and **(D)** egg batches of *I*. *ricinus* females. Bars present means ± standard deviation. IVG^+^, *in vitro* feeding with gentamicin; IVG^−^; *in vitro* feeding without gentamicin; C, control feeding *in vivo* on calves.

The positive effect of gentamicin observed for *in vitro* fed larvae was not seen for *in vitro* fed nymphs. Here, nymphs of the IVG^−^ group had a higher engorgement- and molting proportion (Figure 1B; Supplementary Table S2). The weight of engorged nymphs and unfed females did not significantly differ between IVG^+^ and IVG^−^ (Figures 2A,B; Supplementary Table S2). No significant difference was observed for the engorgement proportion between IVG^+^ nymphs compared to C group nymphs, but nymphs of the IVG^+^ group did have lower engorgement weights, molting proportion and unfed female weights.

#### 3.1.3. Adult feeding

IVG^+^ females did not significantly differ in terms of detachment proportion and detachment weight compared to C group females. However, egg masses and the proportion of viable larvae-producing females were higher for the C group when compared to IVG^+^. Parameters of females did not significantly differ between the IVG^+^ and IVG^−^ (Figures 2C,D; Supplementary Table S3).

### 3.2. Bacterial 16S rRNA sequencing

The DNA concentration from 14/19 unfed female tick samples (IVG^+^: 6/6, IVG^−^: 1/5, C: 7/8) was high enough to pass quality control analysis and was subjected to 16S rRNA amplification and sequencing. The DNA concentration of some extracts from individual ticks, in particular in the IVG^−^ female group without gentamicin, was considered to be too low for 16S rRNA sequencing. DNA extracted from pools of larvae, individual nymphs and males did not pass the quality control of the service provider and were not sequenced. Due to the low sample size, IVG^−^ was excluded for further analysis.

**Figure 3:**
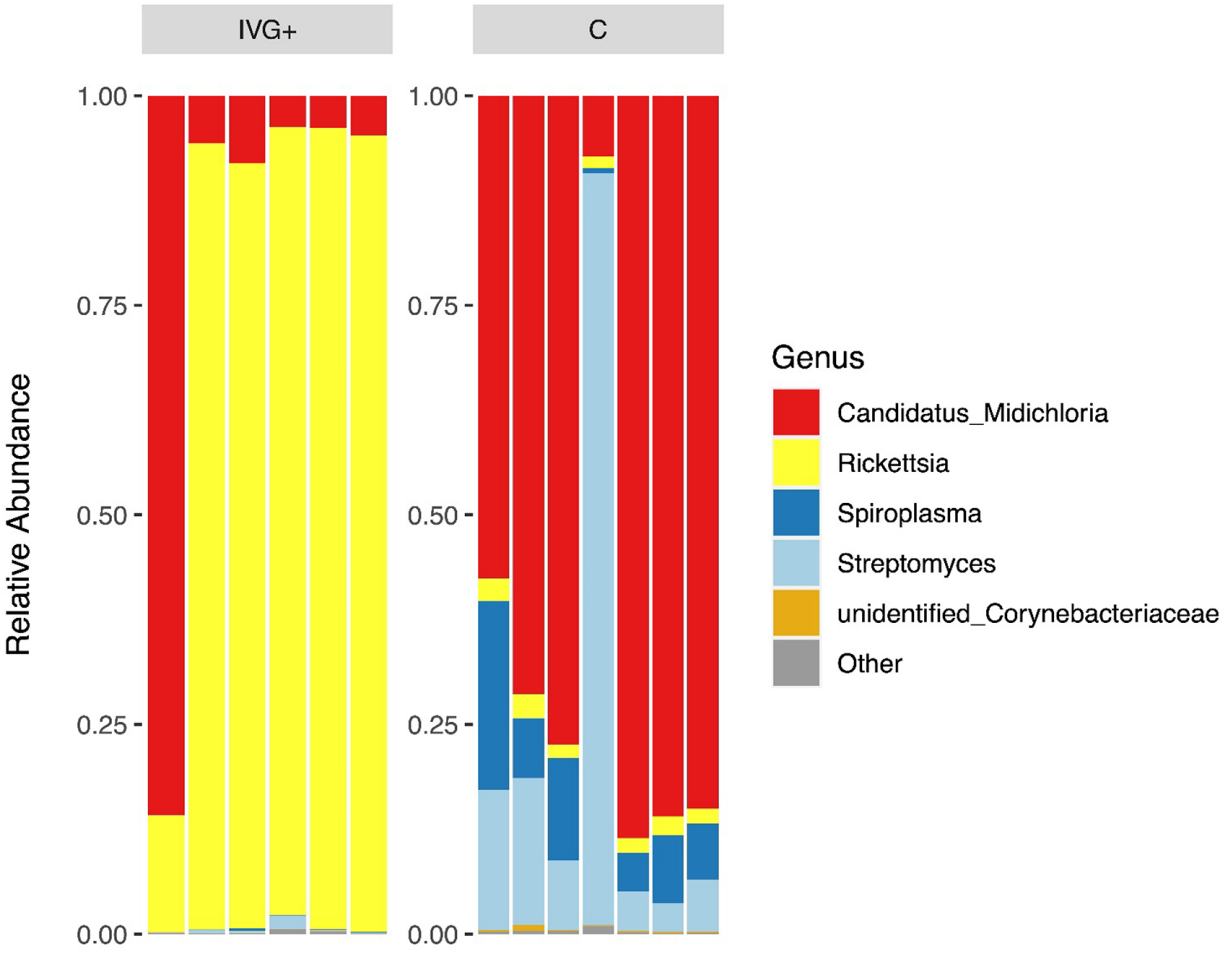
Relative abundances of the top five genera for *in vitro* reared females with gentamicin supplementation (IVG^+^, *n* = 6), and *in vivo* reared females (C, *n* = 7).

In total, 986,684 reads, resulting in 984,005 effective tags after quality filtering and comparison with reference databases were obtained (Supplementary material S1). The mean number of reads per sample in unfed females from the IVG^+^ and C groups were 76,004 (± 11,109) and 74,064 (± 4,493), respectively. Removing contaminants by decontam package resulted in a mean library size of 75,968 (± 11,109) for IVG^+^ and 73,820 (± 4,439) for C group. Overall, females fed as larvae and nymphs on calves had a more diverse microbiome composition compared to females of the IVG^+^ group (Figure 3). All samples except the single IVG^−^ female, contained *Rickettsia* and *M*. *mitochondrii* OTUs. *Streptomyces* (*n* = 13/14) and *Spiroplasma* (*n* = 12/14) were also detected in most samples.

The relative abundance of the bacteria differed between the two groups of females. As shown in Figure 3, the relative abundance of IVG^+^ reared female ticks was dominated by *Rickettsia* OTUs, while females reared on calves had a higher relative abundance of *M*. *mitochondrii* and *Spiroplasma*.

The diversity indices were statistically compared between IVG^+^ and C females (Supplementary Figure S1). In general, there was a tendency for higher diversity means within the C female group, which were significant for the Shannon (Wilcoxon, *p* = 0.008) and the Simpson index (Wilcoxon, *p* = 0.004). Furthermore, PCoA of unweighted and weighted UniFrac and NMDS distances of IVG^+^ females showed a narrow spectrum in comparison to the much broader cluster of control females (Supplementary Figure S2). This bacterial community structure differed between IVG^+^ and C females (weighted UniFrac: df = 1(11), *p* = 0.002, unweighted UniFrac: df = 1 (11), *p* = 0.53).

### 3.3. Sequencing of *Rickettsia* and *Spiroplasma* spp.

The *Rickettsia* species detected by 16S rRNA sequencing was identified as *R*. *helvetica* following sequencing of the *gltA* gene. The *gltA* sequence was 100% identical (499/499 nt) to that of the *R*. *helvetica* C9P9 reference strain (GenBank Accession Number CM001467). The partial *gyrA* sequence of the *Spiroplasma* species showed most identity (506/507 nt, 99.8%) to *Spiroplasma ixodetis* isolated from *I*. *ricinus* (MK267048) and *Ixodes uriae* (MK267049).

### 3.4. Quantitative analyses of bacterial loads

The qPCR data processing was limited to the three main bacterial species detected by 16S rRNA sequencing: *R*. *helvetica*, *M*. *mitochondrii*, and *Spiroplasma* species.

The qPCR data for the three F_0_-larvae batches showed no significant differences for the main bacterial species: all three batches were positive for *M*. *mitochondrii* and *R*. *helvetica*, but negative for *Spiroplasma* (Supplementary Table S4).

**Figure 4:**
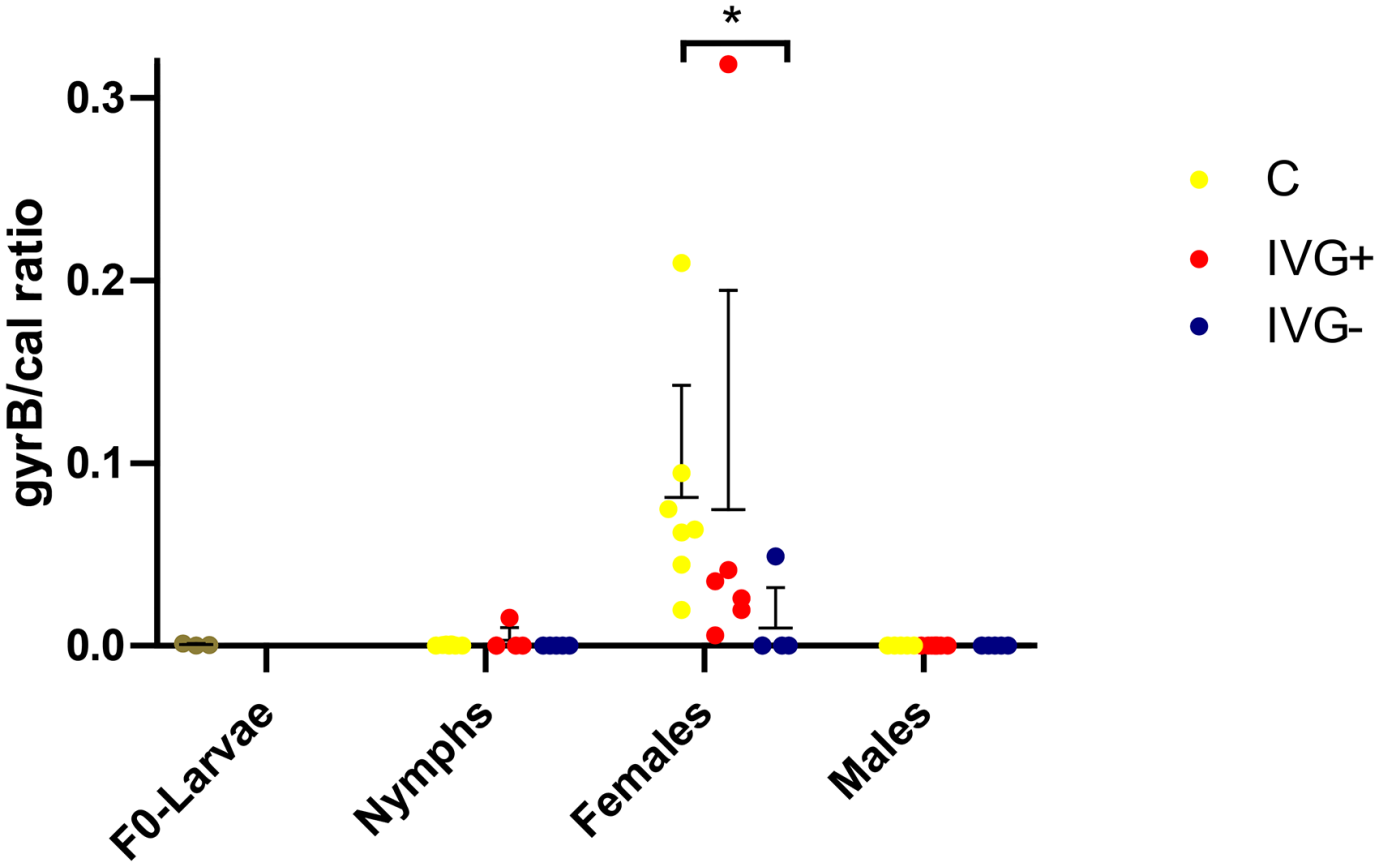
qPCR profiles (relative expression) of *Midichloria mitochondrii*. Each dot represents the relative *gyrB* expression in a single sample. The tick *calreticulin* gene was used as a housekeeping gene. Bars represent mean with the standard deviation shown in error bars. Statistical analysis by Mann–Whitney test performed in Graphpad. IVG^+^, *in vitro* feeding with gentamicin; IVG^−^, *in vitro* feeding without gentamicin; C, control feeding *in vivo* on calves. *p < 0.05.

The *M*. *mitochondrii* abundance was significantly higher in unfed IVG^+^ and C females compared to the corresponding nymphal stages (Mann–Whitney test, *p* = 0.0034 and *p* = 0.0129, respectively), but this was not the case for unfed IVG^−^ females and nymphs (Mann–Whitney test, *p* = 1.0). *Midichloria mitochondrii* was not detected in male ticks. The *M*. *mitochondrii* bacterial loads were significantly higher for the C females compared to IVG^−^ (Mann–Whitney test, *p* = 0.0132; Figure 4).

**Figure 5:**
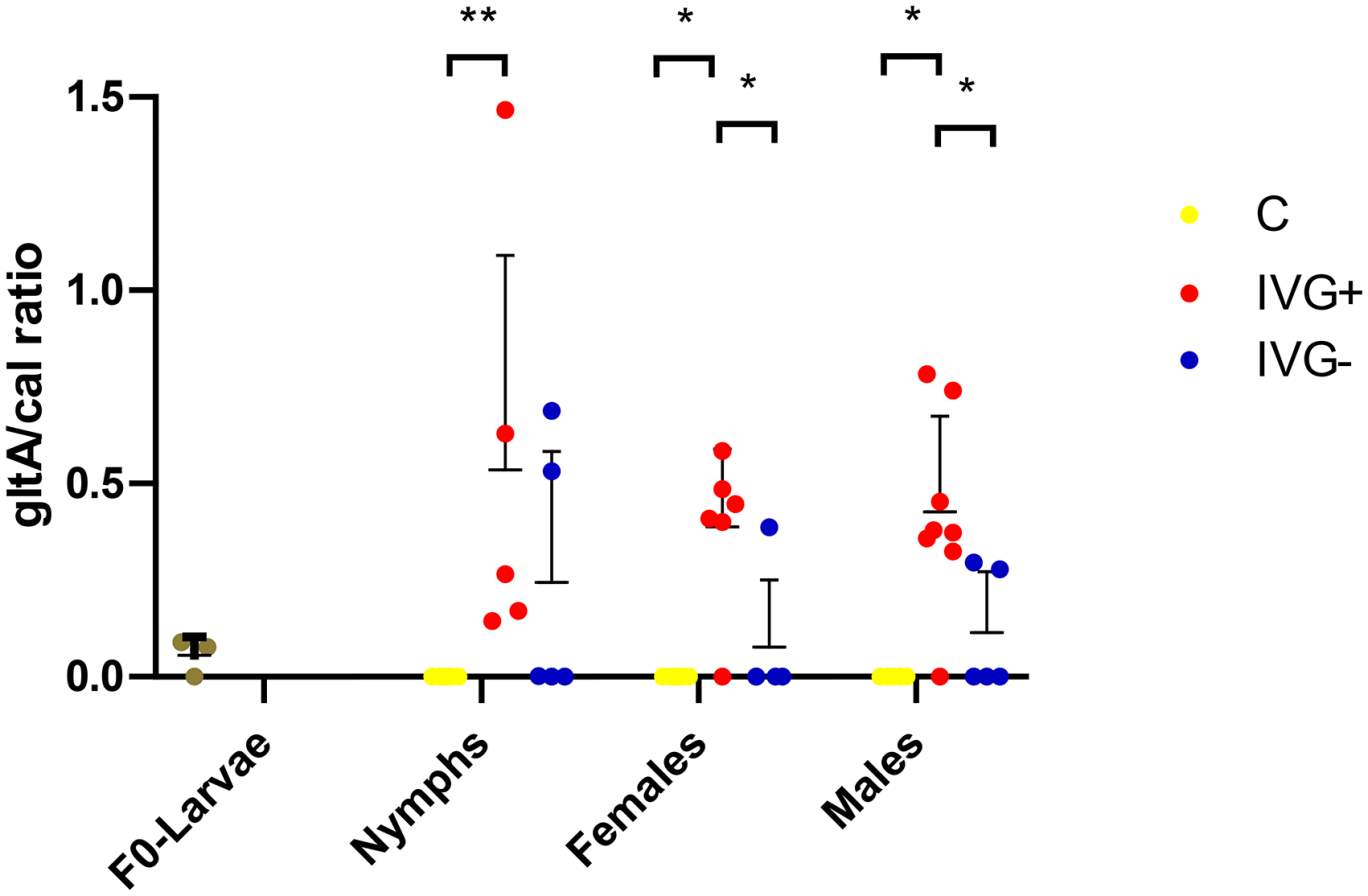
qPCR profiles (relative expression) of *Rickettsia helvetica*. Each dot represents the relative *gltA* expression in a single sample. The tick *calreticulin* gene was used as a housekeeping gene. Bars represent mean with the standard deviation shown in error bars. Statistical analysis by Mann–Whitney test performed in Graphpad. IVG^+^, *in vitro* feeding with gentamicin; IVG^−^, *in vitro* feeding without gentamicin; C, control feeding *in vivo* on calves. *p < 0.05, **p < 0.01.

*Rickettsia helvetica* was also present in all F_0_-larvae groups (Figure 5). The relative number of *R*. *helvetica* bacteria increased in the nymphal IVG^+^ and IVG^−^ ticks, but not in the C group. The relative number of *R*. *helvetica* was significantly higher in IVG^+^ nymphs compared to the C group (Mann–Whitney test, *p* = 0.0043), but did not statistically differ compared to IVG^−^ (Mann–Whitney test, *p* = 0.30), although some IVG^−^ nymphs tested negative for *R*. *helvetica* by qPCR. IVG^+^ and IVG^−^ ticks remained positive as adults at similar levels as nymphs, with significantly higher levels in the IVG^+^ group compared to the C and IVG^−^ group as both females (Mann–Whitney test, *p* = 0.0298 and *p* = 0.0398, respectively) and males (Mann–Whitney test, *p* = 0.0225 and p = 0.0225, respectively).

**Figure 6:**
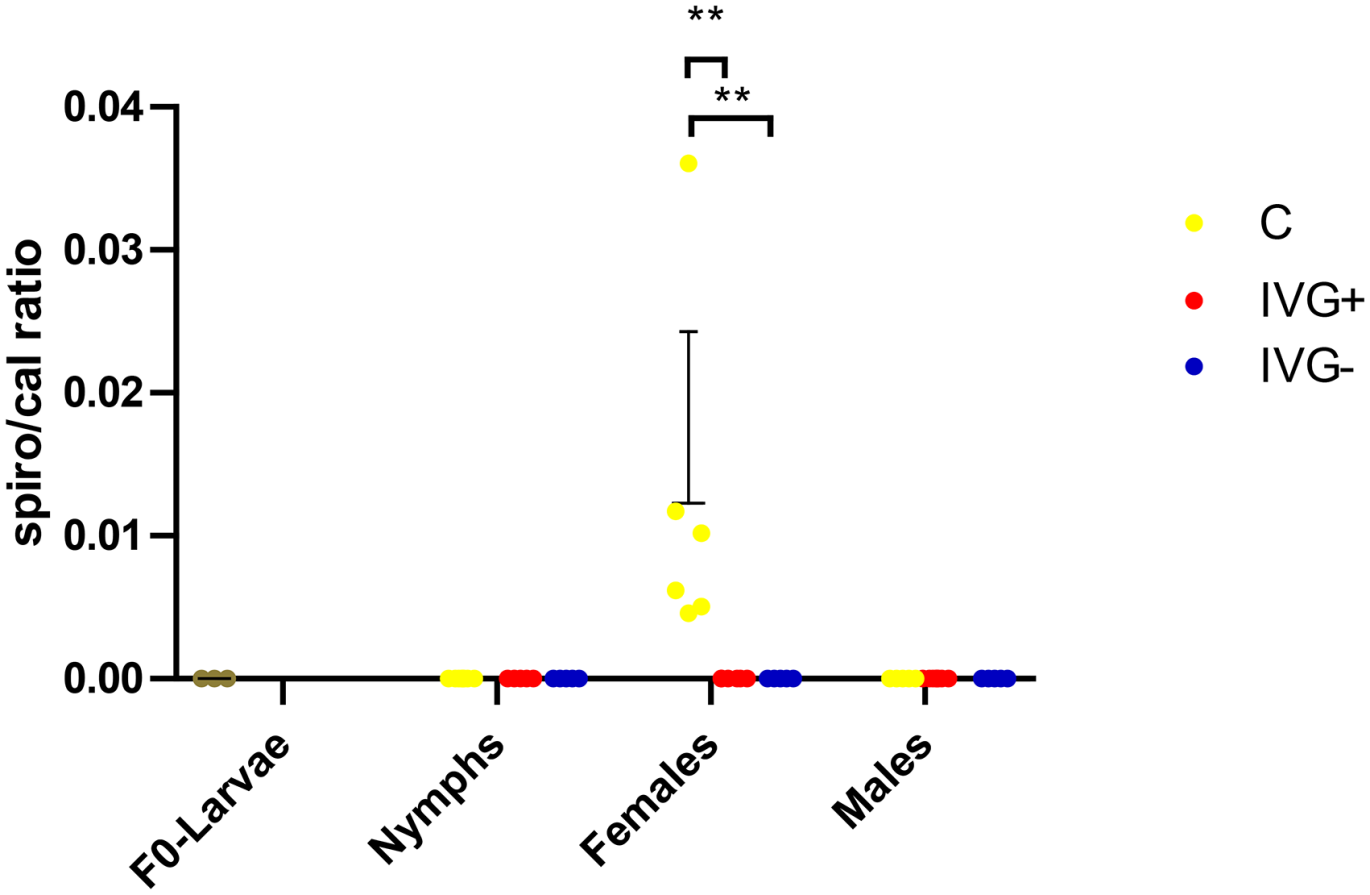
qPCR profiles (relative expression) of *Spiroplasma* spp. Each dot represents the relative *rpoB* expression in a single sample. The tick *calreticulin* gene was used as a housekeeping gene. Bars represent mean with the standard deviation shown in error bars. Statistical analysis by Mann–Whitney test performed in Graphpad. IVG^+^, *in vitro* feeding with gentamicin; IVG^−^, *in vitro* feeding without gentamicin; C, control feeding *in vivo* on calves. **p < 0.01.

Although *Spiroplasma* was detected by 16S rRNA sequencing in most (5/6) IVG^+^ females (albeit in low numbers) and in all C females in higher numbers, they were only detectable by qPCR in the C females (Figure 6).

## 4. Discussion

### 4.1. Effects on tick feeding

In general, ticks from IVG^+^ and IVG^**−**^ showed lower engorgement and detachment weights and a longer feeding duration compared to ticks from the C group. This was in accordance to previous work, where artificially fed ticks were compared to ticks fed on calves (Militzer et al., 2021). The highest proportion of larvae that engorged was found in the IVG^+^ group. It should be noted that although all life stages of *I*. *ricinus* can feed on large ruminants, they are not commonly used as experimental animals for the feeding of *I*. *ricinus* larvae, so there is little information available on the feeding efficacy of larvae on cattle (Jaenson et al., 1994; Hofmeester et al., 2016; Levin and Schumacher, 2016). This may explain the limited engorgement proportion observed for the control group larvae. We nonetheless preferred to use only cattle as hosts or blood source for all experiments to reduce variation between experiments, as the source of the blood meal was previously shown to affect tick feeding and molting (Koch and Hair, 1975; Brunner et al., 2011). The molting proportions were lower for IVG^+^ and IVG^−^ fed larvae compared to the C larvae, which might be explained by their significantly lower engorgement weight. Previous studies on *Amblyomma americanum* showed that the molting success of larvae and nymphs was associated to their engorgement weight; ticks that failed to reach a “critical” engorgement weight did not molt (Koch, 1986). Similar results were observed for *Rhipicephalus sanguineus* nymphs (Ben-Yosef et al., 2020).

The supplementation of the blood meal with gentamicin and B vitamins resulted in higher engorgement or detachment weights, in particular for females (IVG^+^). In general, female ticks typically have a longer feeding duration than juvenile ticks, which increases the risk of bacterial contamination of the blood meal. This may have led to a reduction in the blood meal quality and prevented the ticks from fully engorging. Juvenile *I*. *ricinus* ticks did feed successfully without antibiotics, corroborating results of other studies where antibiotics were also omitted from the blood meal, for instance to prevent possible interference of antibiotics with pathogen acquisition or transmission (e.g., Waladde et al., 1993; Koci et al., 2018; Korner et al., 2020). It is important to note here that data on the artificial feeding of consecutive hard tick life stages is very limited (Kuhnert et al., 1995; Militzer et al., 2021). A possible cumulative effect of antibiotics on ticks and their endosymbionts cannot be ruled out and need to be studied further.

### 4.2. Effects on the microbiome diversity

In our study, the microbiome of female ticks fed as larvae and nymphs on the ears of calves was more diverse than that of the IVG^+^ group and possibly from that of the IVG^−^ group, for which only limited 16S rRNA sequencing data was available. This difference in variety could be explained by the feeding process: C ticks fed on calves were exposed to a wider variety of bacteria, for instance from the microbiome of the bovine skin and cerumen, compared to the IVG^+^ and IVG^−^ groups that were fed in a relatively sterile laboratory environment. They may therefore have acquired a more diverse set of bacteria from their environment by oral or cuticular routes compared to the IVG^+^ ticks. In addition, the microbiome variety could have been reduced by the exposure of IVG^+^ ticks to gentamicin, but the lack of sufficient data for the IVG^−^ group prevents the drawing of definitive conclusions in this regard.

Interestingly, most of the IVG^+^ and IVG^−^ females (*n* = 6/7) had a high relative abundance of *R*. *helvetica* and low relative abundances of *M*. *mitochondrii* and *Spiroplasma* spp. This was in contrast to the C females, where most ticks (*n* = 6/7) showed a high relative abundance of *M*. *mitochondrii*, a finding supported by qPCR data. However, when C and IVG^+^ groups were directly compared, the difference was not significant. This was mostly due to one sample of the IVG^+^ group, which had an exceptionally high *M*. *mitochondrii/calreticulin* ratio of 0.318, corroborating its 16S rRNA sequencing result (Figures 3, 4).

*Midichloria mitochondrii* is abundant in various tick species collected from the field, with a reported prevalence ranging from 54.8 to 100% in *I*. *ricinus* females (Lo et al., 2006; Sassera et al., 2006; Duron et al., 2017; Garcia-Vozmediano et al., 2021). A prevalence below 100% may suggest that the symbiosis is not obligatory for *I*. *ricinus* to survive, or that other symbionts take over this role when *Midichloria* is absent (Krawczyk et al., 2022a). The number of *Midichloria* was shown to decrease after the molt of *I*. *ricinus* and increase during blood meal intake, suggesting that it may be relevant for tick development, for instance by providing the tick with essential nutrients that are missing in the blood meal (Sassera et al., 2008; Olivieri et al., 2019). In females, *Midichloria* have mainly been found in ovaries, which ensures its maternal transition (Epis et al., 2013; Olivieri et al., 2019). In our study, the analysis for *M*. *mitochondrii* was performed in unfed ticks, i.e., prior to feeding, which may explain the relatively low bacterial loads found compared to previous reports (Sassera et al., 2008). Of note, the laboratory tick colony originated from ticks flagged in a study site that was previously shown to have a low *Midichloria* prevalence (36.4%) in nymphs (Garcia-Vozmediano et al., 2021). Furthermore, *M*. *mitochondrii* was not detected in male ticks, corroborating previous reports describing a low *Midichloria* abundance in males (Lo et al., 2006; Sassera et al., 2008; Lejal et al., 2020; Garcia-Vozmediano et al., 2021). Future male nymphs have also been reported to have lower *Midichloria* loads than future female-nymphs (Epis et al., 2013; Daveu et al., 2021). Since differentiation between future male and future female was not possible at the time of testing, this may also have influenced the detection of *M*. *mitochondrii* in nymphs in our study.

*Ixodes* spp. ticks have been shown to harbor several *Rickettsia* species (Kurtti et al., 2015; Hajduskova et al., 2016; Duron et al., 2017; Nováková and Šmajs, 2018). So far, *R*. *helvetica*, *Candidatus* R. mendelii, *R*. *monacensis*, *R*. *raoultii*, *R*. *slovaca*, and *Candidatus* R. thierseensis have been detected in *I*. *ricinus* ticks, with *R*. *helvetica* being the most common *Rickettsia* species found (Simser et al., 2002; Hajduskova et al., 2016; Schötta et al., 2017, 2020). The 16S rRNA sequencing results and additional *gltA* sequencing suggest that the *I*. *ricinus* ticks used for this study only contained *R*. *helvetica*. As the sequence of the 16S rRNA V3-V4 region of *R*. *helvetica* differs from that of the other *Rickettsia* species that have been associated with *I*. *ricinus*, the presence of these *Rickettsia* spp. in ticks used for this study is not plausible.

*Rickettsia* spp. are gram-negative intracellular alpha-proteobacteria that can be categorized in several groups (Salje, 2021). *Rickettsia helvetica* belongs to the spotted fever group (SFG), a group that contains several pathogenic species such as *R*. *rickettsi* and *R*. *conorii*, the causal agents of Rocky Mountain spotted fever and Mediterranean spotted fever, respectively, but also contains *Rickettsia* species of undetermined pathogenicity. Even though previous literature reported the detection of *R*. *helvetica* in a small number of diseased humans, disease causation has not been convincingly demonstrated and the pathogenicity of *R*. *helvetica* remains to be determined (Nilsson et al., 1999, 2010; Azagi et al., 2020).

qPCR results showed that *R*. *helvetica* was the predominant bacterial species in IVG^−^ and IVG^+^ groups, confirming the 16S rRNA sequencing data for IVG^+^ (Figures 3, 5). This was a striking finding, as all three F_0_-larvae batches with which the study started contained similar amounts of *R*. *helvetica* (Figure 5 and Supplementary Table S4). This suggests that artificial feeding led to a positive selection for *R*. *helvetica* in the majority of the analyzed samples. We hypothesize that this may have been caused by interactions between *R*. *helvetica* and the microbiome, which was less varied compared to the C group, leading to a dysbiosis that could have facilitated *R*. *helvetica* colonization of the ticks. Interestingly, a recent study described a significant reduction in the microbiota diversity in *I*. *ricinus* nymphs collected from humans that were infected with *R*. *helvetica* (Maitre et al., 2022). The authors hypothesized that *R*. *helvetica* may modulate the tick microbiome to facilitate colonization whereas our results raise the question if a high *R*. *helvetica* abundance could not actually be the result of a reduced tick microbiome diversity. The presence of *R*. *helvetica* in bovine blood used as a blood meal source could be an alternative explanation for the increased *R*. *helvetica* abundance. Although there are no reports on the detection of *R*. *helvetica* in bovine blood, it has been detected in the blood of other ruminants such as domestic goats (*Capra hircus*), roe deer (*Capreolus capreolus*) and sika deer (*Cervus nippon yeiensis*; Inokuma et al., 2008; Stefanidesova et al., 2008; Rymaszewska, 2018). However, the original blood samples used for the artificial feeding were not available anymore to test this hypothesis. Although blood collected from the same donor cattle several months after the use of their blood for artificial feeding of larvae tested negative for the presence of *R*. *helvetica* DNA by PCR (results not shown), this alternative hypothesis cannot be fully excluded. The observed high abundance of *R*. *helvetica* in IVG^+^ and IVG^−^ ticks could be useful for experimental studies in which a high pathogen abundance in ticks is advantageous. On the other hand, it also shows that the composition of the tick microbiome should be taken into account in ATFS acquisition and transmission studies, as ATFS itself may have a direct effect on the tick microbiome and tick-borne pathogen abundance. Successful colonization of *Ixodes* ticks with the causal agent of Lyme Borreliosis, *Borrelia burgdorferi sensu stricto*, has for instance been associated with a higher microbiome diversity (Narasimhan et al., 2017; Sperling et al., 2020). This should be considered in the experimental design of ATFS acquisition and transmission models for this pathogen.

A third species that was particularly abundant in the C females was *Spiroplasma* (Figure 6). *Spiroplasma ixodetis* is considered to be a facultative symbiont and has previously been detected in *I*. *ricinus* ticks, but its effect on ticks has not been clarified yet (Duron et al., 2017; Lejal et al., 2021). *Spiroplasma* spp. in *Ixodes* ticks are thought to maternally inherited (Beliavskaia et al., 2021) and although we did detect *Spiroplasma* OTUs in both the IVG^+^ and C females, we could not detect *Spiroplasma* DNA by qPCR in the F_0_-larvae to confirm transovarial transmission. However, this may also have been caused by limitations in the sensitivity of the used qPCR for the detection of *Spiroplasma*. A previous study on the microbiome of *I*. *ricinus* nymphs collected from the vegetation near Paris, France, showed a decreased abundance of *Spiroplasma* in *Rickettsia-*positive samples (Lejal et al., 2021), which corroborates with our findings where *Spiroplasma* was not detected by qPCR in ticks with a high *R*. *helvetica* abundance. This negative association is suggestive of competition or niche partitioning between *Spiroplasma* and *R*. *helvetica* (Krawczyk et al., 2022a,b).

Endosymbionts such as *Midichloria* are thought to play an important role in tick biology by providing essential B vitamins to ticks (Duron et al., 2018; Duron and Gottlieb, 2020). The most common bacteria associated with providing essential B vitamins other than *Midichloria* are *Coxiella*-like endosymbionts, *Francisella*, and some *Rickettsia* spp (Hunter et al., 2015; Duron et al., 2017). Although the production of a core set of B vitamins (biotin, riboflavin and folate) is usually associated with a single nutritional symbiont for each tick species (Duron et al., 2017), it was recently suggested that in some tick species a dual endosymbiosis occurs whereby a second endosymbiont provides B vitamin components that the other endosymbiont cannot produce (Buysse et al., 2021). Previous analyses showed that the genome of *M*. *mitochondrii* contains genes for the synthesis of biotin and folate, but does not seem to have all genes required for the synthesis of riboflavin (Buysse et al., 2021). It leaves the question from which source *I*. *ricinus* obtains riboflavin, provided that the levels found in blood are insufficient. The genome of *R*. *helvetica* does not have a functional riboflavin pathway and it would be interesting to examine if the genomes of other bacteria associated with *I*. *ricinus*, such as *S*. *ixodetis*, *Rickettsiella* or perhaps *Streptomyces* species would have functional B vitamin synthetic pathways. If so, this might also explain how *I*. *ricinus* ticks in which *M*. *mitochondrii* is absent obtain essential B vitamin components. We also observed a negative association between *Midichloria* and *R*. *helvetica*. The same negative association was found in a previous study in which nearly 14,000 questing *I*. *ricinus* nymphs were screened by qPCR for tick-associated microorganisms (Krawczyk et al., 2022b). In contrast, other studies reported a positive association between *Midichloria* and *Rickettsia* spp., both in questing ticks and ticks collected from humans (Budachetri et al., 2018; Lejal et al., 2021; Maitre et al., 2022). These contrasting results may in part be explained by factors found to be of influence the microbiome composition of ticks that differed between the studies, such as environmental temperature and the identity of hosts on which the ticks fed (Swei and Kwan, 2017; Thapa et al., 2019).

A major limitation of this study is the low sample size for the 16S rRNA sequencing, due to low DNA yields. It has previously been reported that the extraction of DNA from single *I*. *scapularis* ticks and samples with a low biomass may result in low yields (Ammazzalorso et al., 2015). To overcome the lack of 16S rRNA sequencing data for the juvenile life stages, additional qPCRs were performed for larvae and nymphs, in which constant results for the tick *calreticulin* gene were obtained (Supplementary Table S4). It is known that low biomass samples are at a higher risk for contamination sequences than higher biomass samples (Salter et al., 2014; Eisenhofer et al., 2019; Lejal et al., 2020). Pooling of ticks would have been an alternative to increase DNA yields for sequencing and to have robust samples against biases and contamination challenges. Although pooling of ticks gives only limited insights in microbial communities and diversities, it could have been an alternative in combination with qPCR (Krawczyk et al., 2022a). Future studies should take these observations into account.

Another limitation of this study is the relatively low number of ticks that could consecutively be fed from the larval to the adult stage. The resulting sample size was too low to conduct further statistical analyses on eggs and F_1_-larvae. The absence of B vitamin components in the blood meals offered to the *in vitro* fed larvae and nymphs could have negatively influenced tick fitness and development at these stages. The optimal dose of B vitamin supplementation and its effect on the larvae and nymphs should be examined in more detail in future studies aimed at optimizing the artificial feeding of *I*. *ricinus*.

In conclusion, we examined the microbiome of *I*. *ricinus* under different experimental conditions by feeding all consecutive life stages of *I*. *ricinus* by ATFS on blood meals with (IVG^+^) or without gentamicin (IVG^−^) and comparing the feeding parameters to those of ticks fed simultaneously on calves (C). The tick microbiome composition was studied by 16S rRNA sequencing and qPCRs for *M*. *mitochondrii*, *R*. *helvetica*, and *Spiroplasma* spp. The results showed a shift of the ticks’ microbiome, with the symbiont *M*. *mitochondrii* being the dominant genus for females fed as larvae and nymphs on calves and *R*. *helvetica* being the most abundant bacteria in females that were fed as juveniles *in vitro*. IVG^−^ females showed significant lower loads of *M*. *mitochondrii* compared to the other groups. *Spiroplasma* spp. loads also differed: while exclusively detected in C female ticks by qPCR, 16S rRNA sequencing results also showed low relative abundances in IVG^+^ females. Collectively, the results showed that the employed feeding techniques affect the fecundity and microbiome composition of ticks, with a decreased microbiome diversity in artificially fed ticks fed on blood supplemented with gentamicin. These effects should be taken into account in studies employing ATFS.

## Data availability statement

The data presented in the study are deposited in the NCBI BioProject repository, accession number PRJNA905798.

## Ethics statement

All animal experiments were approved by the regional authorities for animal experiments (LaGeSo, Berlin, 0387/17).

## Author contributions

AN, NM, and SPS conceptualized this study. NM carried out methodology. AN and NM performed the formal analysis and wrote the original draft. All authors read and approved the final manuscript.

## Funding

This study was funded by the German Federal Ministry of Education and Research (BMBF, grant number 01KI1720) as part of the Junior Research Group “Tick-borne Zoonoses.” This research was further funded by Deutsche Forschungsgemeinschaft (German Research Foundation, DFG) through the Research Training Group GRK 2046 “Parasite Infections: From experimental models to natural systems” (NM and SPS associated Ph.D. candidates/AN Senior Researcher).

## Conflict of interest

The authors declare that the research was conducted in the absence of any commercial or financial relationships that could be construed as a potential conflict of interest.

## Publisher’s note

All claims expressed in this article are solely those of the authors and do not necessarily represent those of their affiliated organizations, or those of the publisher, the editors and the reviewers. Any product that may be evaluated in this article, or claim that may be made by its manufacturer, is not guaranteed or endorsed by the publisher.

## Footnotes

^1^ http://www.premierbiosoft.com/netprimer/

^2^ http:///www.mbio.ncsu.edu/BioEdit/bioedit.html
