# Supplementary material for: Changes in the Ixodes ricinus microbiome associated with artificial tick feeding
Source: Front Microbiol. 2023 Jan 10;13:1050063. doi: 10.3389/fmicb.2022.1050063 (PMC9871825; doi:10.3389/fmicb.2022.1050063)
Supplement: Supplementary file 1 [file Data_Sheet_1.docx]

**
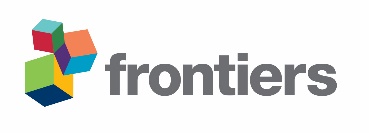
**

Supplementary Material

# Supplementary Figures


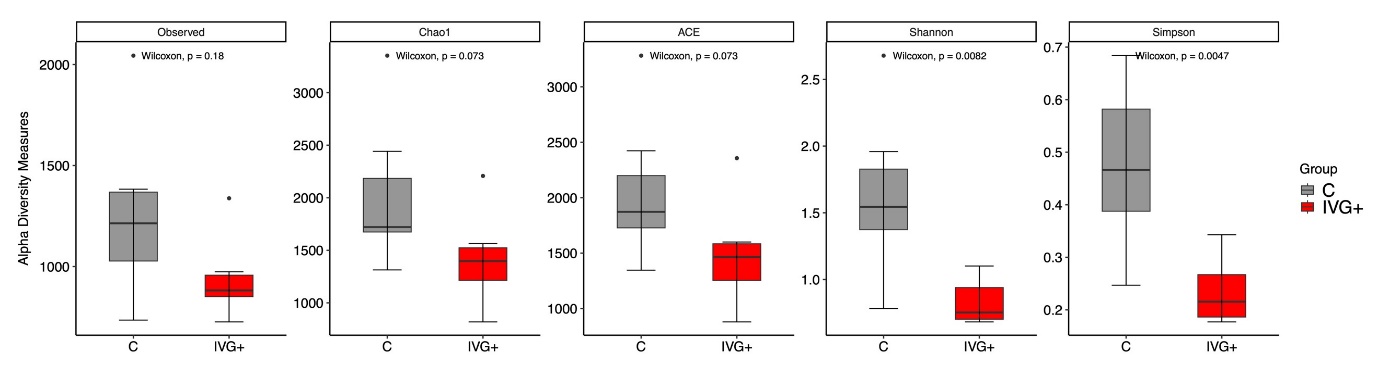


**Supplementary Figure 1**: Impact on alpha diversity indices of *in vitro* reared females with gentamicin treatment (IVG^+^, n=6) to *in vivo* reared female ticks (C, n=7): Observed species, Chao1, ACE, Shannon and Simpson index. For statistical significances (p-value) a Wilcoxon-test was performed.

**
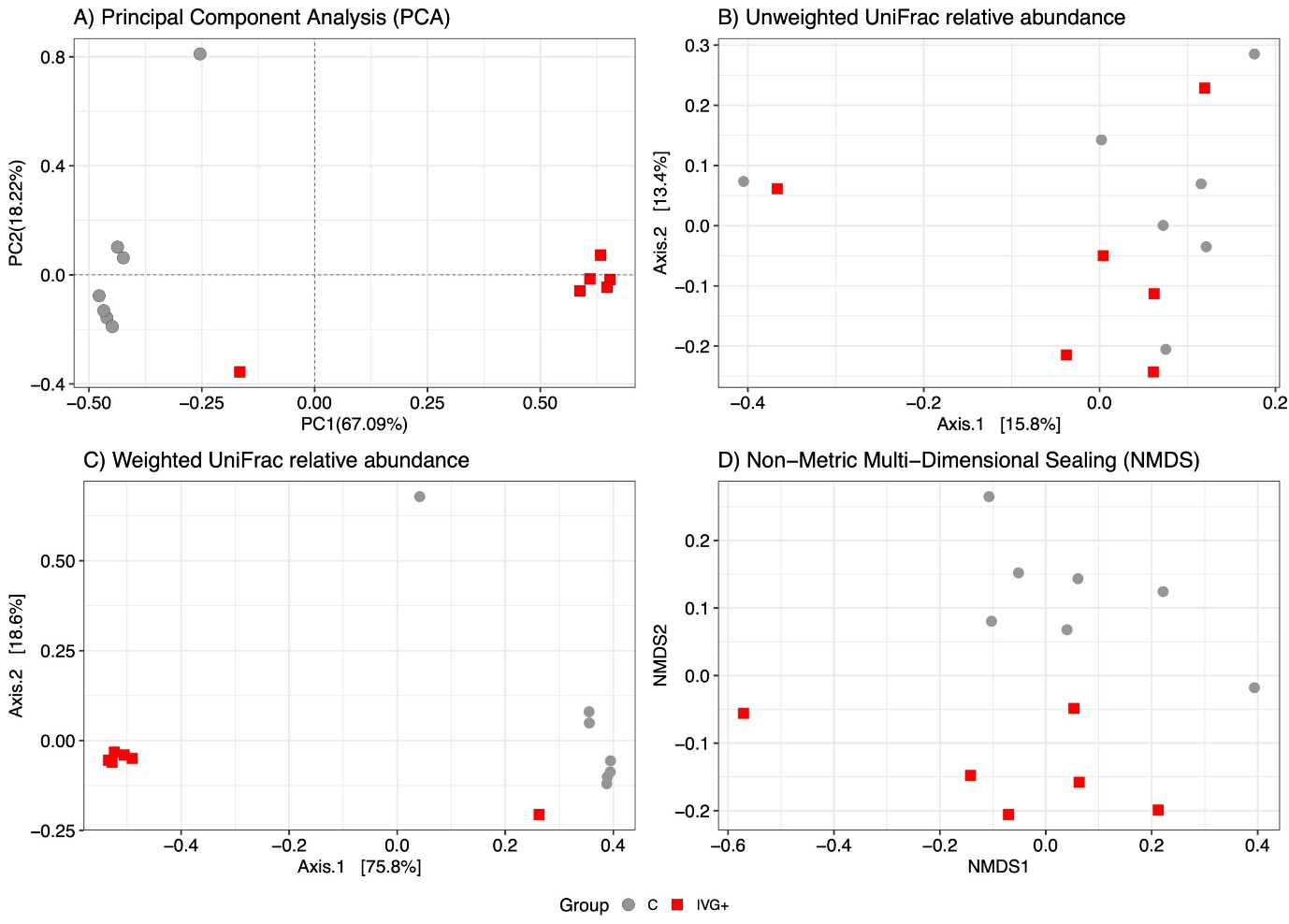
**

**Supplementary Figure 2.** Beta-diversity means of *in vitro* with gentamicin reared female tick (IVG^+^, n=6), *in vitro* without gentamicin treatment female ticks (IVG^-^, n=1), and *in vivo* reared female ticks (C, n=7): A) Principal component analysis (PCA), B) principal coordinate analysis (PCoA) of B) unweighted and C) weighted UniFrac distances, and D) Non-metric Multi-Dimensional Sealing are presented.

**Supplementary Table 1.** Data on *in vitro* (with gentamicin IVG^+^ and without IVG^-^) and *in vivo* (C) feeding of larvae ticks presented as mean ± standard deviation (SD) and the 95% confidence interval (CI) or coefficient of variation (CV). Proportions analyzed by Z-test, analysis on engorgement weight and feeding duration analyzed by duration by Mann Whitney U test (MWU). p-value, df = degrees of freedom, χ2 = chi-square. *Larvae: per feeding unit IVG^+^ (n= 6), IVG^-^ (n= 13), *in vivo* per feeding experiment (n=1) **Larvae: per weighted larvae batch IVG^+^ (n= 4), IVG^-^ (n= 3) or per experiment *in vivo* (n= 1).

| **Parameter** | ***in vitro*** | | ***in vivo***  **(C)** | **Statistical Analyses**  (p-Value, df = Degrees of Freedom, χ^2^ = Chi-square) | | |
| --- | --- | --- | --- | --- | --- | --- |
|  | **Without gentamicin**  **(IVG^-^)** | **With gentamicin**  **(IVG^+^)** |  | **IVG^-^ to IVG^+^** | **IVG^-^ to C** | **IVG^+^ to C** |
| Estimated number of larvae used | 1505 | 709 | 1160 |  | | |
| Engorgement (%) | 25  (CI: 22 – 27) | 61  (CI: 56 – 64) | 54  (CI: 50 – 57) | df= 1,  χ^2^= 260.2,  p< 0.0001 | df= 1,  χ^2^= 229.5,  p< 0.0001 | df= 1,  χ ^2^= 7.79,  p= 0.0052 |
| Mean duration of feeding experiment (days)* | 5.8 ± 1.4  (CV: 3.7 – 4.4) | 6.3 ± 0.5  (CV: 3.3 – 4.4) | 4 | MWU,  W= 31,  p= 0.652 |  |  |
| Mean engorgement weight (mg)** | 0.358 ± 0.09 (CI: 0.135 – 0.582) | 0.4 ± 0.01  (CI: 0.389 – 0.41) | 0.529 | MWU,  W= 31,  p= 0.652 |  |  |
| Molting per engorged tick (%) | 29  (CI: 25 – 34) | 38  (CI: 34 – 44) | 71  (CI: 67 – 75) | df= 1,  χ^2^= 7.8,  p= 0.0051 | df= 1,  χ^2^= 165.2,  p< 0.0001 | df= 1,  χ^2^= 105.5,  p< 0.0001 |

**Supplementary Table 2:** Data on *in vitro* (with gentamicin IVG^+^ and without IVG^-^) and *in vivo* (C) feeding of nymphs presented as mean ± standard deviation (SD) and the 95% confidence interval (CI) or coefficient of variation (CV). Proportions analyzed by Z-test, analysis on feeding duration and engorgement weight by Mann Whitney U test (MWU), and analysis on molted female weight by t-test. p-value, df = degrees of freedom, χ2 = chi-square. F: female ticks, M: male ticks. *Nymphs: per feeding unit IVG^+^ (6 x n= 20, 1x n= 16), IVG^-^ (4 x n= 20, 1 x n= 16), *in vivo* per feeding experiment (4 x n= 20, 1 x n= 16).

| **Parameter** | ***in vitro*** | | ***in vivo***  **(C)** | **Statistical Analyses**  (p-value, df = Degrees of Freedom, χ^2^ = Chi-square) | | |
| --- | --- | --- | --- | --- | --- | --- |
|  | **Without gentamicin (IVG^-^)** | **With gentamicin**  **(IVG^+^)** |  | **IVG^-^ to IVG^+^** | **IVG^-^ to C** | **IVG^+^ to C** |
| Number of fed ticks | 96 | 136 | 200 |  | | |
| Engorgement (%) | 93  (CI: 85 – 96) | 70  (CI: 61 – 77) | 79  (CI: 72 – 84) | df= 1,  χ^2^= 16.5,  p< 0.0001 | df= 1, χ^2^= 7.9, p= 0.005 | df= 1,  χ^2^= 3.2,  p= 0.075 |
| Mean duration until feeding experiment (days)* | 10 ± 1.2  (CV: 12.2) | 8 ± 1.1  (CV: 13.7) | 6 ± 0 | MWU,  W= 27,  p= 0.032 |  |  |
| Mean engorgement weight (mg) | 2.88 ± 0.83  (CI: 2.7 – 3.05) | 2.92 ± 0.83  (CI: 2.74 – 3.09) | 3.59 ± 0.92 (CI: 3.45 – 3.73) | MWU,  W= 4324,  p= 0.793 | MWU,  W= 3980,  p< 0.0001 | MWU,  W= 4331,  p< 0.0001 |
| Female proportion (%) | 57  (CI: 44 – 68) | 48  (CI: 34 – 62) | 56  (CI: 47 – 64) | df= 1,  χ^2^= 0.57,  p= 0.4487 | df= 1,  χ^2^ <0.0001, p= 1 | df= 1,  χ^2^= 0.7, p= 0.419 |
| Molting per engorged tick (%) | 73  (CI: 63 – 81) | 51  (CI: 40 – 60) | 82  (CI: 75 – 87) | df= 1,  χ^2^= 8.9, p= 0.003 | df= 1,  χ^2^= 2.4, p= 0.125 | df= 1,  χ^2^= 27.2, p< 0.0001 |
| Weight of molted adults (mg) | F: 1.29 ± 0.32  (CI: 1.19 – 1.4)  M: 0.78 ± 0.17  (CI: 0.71 – 0.84) | F: 1.37 ± 0.38  (CI: 1.2 – 1.53)  M: 0.82 ± 0.17 (CI: 0.74 – 0.89) | F: 1.7 ± 0.2 (CI: 1.65 – 1.75)  M: 1.0 ± 0.1 (CI: 1.0 – 1.1) | F: df= 40 p= 0.445 | F: df= 52 p< 0.0001 | F: df= 26,  p< 0.0001 |

**Supplementary Table 3:** Data on *in vitro* (with gentamicin IVG^+^ and without IVG^-^) and *in vivo* (C) feeding of adult female ticks presented as mean ± standard deviation (SD) and the 95% confidence interval (CI) or coefficient of variation (CV). Due to the failure to feed adults *in vivo* in July 2020, adult *in vivo* data had to be conducted using ticks from a new tick batch (also fed as larvae and nymphs on calves) in parallel to *in vitro* feedings. Proportions analyzed by Z-test, analysis on durations by Mann Whitney U test (MWU), and analysis on weights and masses by t-test. p-value, df = degrees of freedom, χ2 = chi-square. F: female ticks, M: male ticks.

| **Parameter** | ***in vitro*** | | ***in vivo***  **(C)** | **Statistical Analyses**  (p-value, df = Degrees of Freedom, χ^2^ = Chi-square) | | |
| --- | --- | --- | --- | --- | --- | --- |
|  | **Without gentamicin (IVG^-^)** | **With gentamicin (IVG^+^)** |  | **IVG^-^ to IVG^+^** | **IVG^-^ to C** | **IVG^+^ to C** |
| Number of fed ticks (females : males) | 25 : 20 | 10 : 10 | 100 : 100 |  | | |
| Detachment >55mg (%) | 40 (CI: 23 – 59) | 70 (CI: 39 – 89) | 87 (CI: 79 – 92) | df= 1,  χ2= 1.4,  p= 0.108 | df= 1,  χ^2^= 25.4,  p<0.0001 | df= 1,  χ^2^= 2.1,  p=0.146 |
| Mean detachment weight (mg) | 129 ± 53  (CI: 91 – 167) | 172 ± 97  (CI: 81 – 262) | 233 ± 74  (CI: 217 – 249) | df= 8,  p= 0.322 | df= 13,  p< 0.0001 | df= 6,  p= 0.152 |
| Mean duration until detachment (days) | 17.3 ± 6.2  (CV: 36) | 15.3 ± 6.8  (CV: 44.2) | 6.9 ± 1.2  (CV: 17.2) | MWU, W= 29, p= 0.587 | MWU,  W= 856.5,  p< 0.0001 | MWU, W= 600, p< 0.0001 |
| Oviposition (%) | 50 (CI: 23–76) | 43 (CI: 15 – 75) | 86 (CI: 77 – 92) | df= 1,  χ2= 0.08, p= 0.772 | df= 1,  χ2= 8.13,  p= 0.004 | df= 1,  χ2= 8.62, p= 0.003 |
| Mean pre-oviposition duration (days) | 23.2 ± 14.9  (CV: 64.3) | 21.3 ± 12.7  (CV: 53) | 13.9 ± 1.8  (CV: 13.3) | MWU,  W= 7,  p= 1 | MWU,  W= 266,  p= 0.094 | MWU,  W= 166, p= 0.236 |
| Mean egg mass (mg) | 23 ± 12  (CI: 4 – 41) | 55 ± 60  (CI: 0 – 204) | 132 ± 41  (CI: 122 – 141) | df= 2, p= 0.449 | df= 8,  p< 0.0001 | df= 2,  p= 0.156 |
| Larvae producing females (%) | 10 (CI: 2 – 29) | 20 (CI: 5 – 51) | 71 (CI: 61 – 79) | df= 1, χ^2^= 0.7, p= 0.416 | df=1,  χ^2^= 27.4, p< 0.0001 | df= 1,  χ^2^= 10.6, p= 0.001 |

**Supplementary Table 4.** Results of each developmental stage of mean DNA copy numbers (cal= calreticulin gene (*I. ricinus*), gyrB= Ca. *M*. mitochondrii, gltA= *R. helvetica*, rpoB= *Spiroplasma* spp.) by qPCR; n= sample size per group, SD= standard deviation, IVG^+^ = *in vitro* with gentamicin treatment, IVG^-^ = *in vitro* without gentamicin, C= *in vivo* ticks fed on calves.

| **copy numbers** | | **cal copy number** | | | **gyrB copy number** | | | **gltA copy number** | | | **rpoB copy number** | | |
| --- | --- | --- | --- | --- | --- | --- | --- | --- | --- | --- | --- | --- | --- |
| **Stage** | **Group** | **Mean** | **n** | **SD** | **Mean** | **n** | **SD** | **Mean** | **n** | **SD** | **Mean** | **n** | **SD** |
| F_0_-Larvae |  | 13143 | 3 | 3701 | 8,0 | 3 | 7,69 | 841,00 | 3 | 730 | 0 | 3 |  |
| Nymphs | IVG- | 5823 | 5 | 1188 | 0,2 | 5 | 0,42 | 1556,00 | 5 | 2275 | 0 | 5 |  |
|  | IVG+ | 74454 | 5 | 94153 | 28,8 | 5 | 57,5 | 14429,00 | 5 | 12969 | 0 | 5 |  |
|  | C | 6959 | 8 | 1059 | 1,9 | 8 | 2,23 | 0,51 | 8 | 0,46 | 0 | 8 |  |
| Females | IVG- | 251209 | 5 | 69653 | 2367,0 | 5 | 5293 | 18637,00 | 5 | 41673 | 0 | 5 |  |
|  | IVG+ | 249803 | 6 | 155306 | 11434,0 | 6 | 9580 | 103945,00 | 6 | 74715 | 0 | 6 |  |
|  | C | 240673 | 7 | 85157 | 14425,0 | 8 | 9755 | 0,20 | 8 | 0,2 | 2421 | 7 | 2019 |
| Males | IVG- | 15670 | 5 | 4738 | 0,0 | 5 |  | 1644,00 | 5 | 2327 | 0 | 5 |  |
|  | IVG+ | 13311 | 8 | 4038 | 0,0 | 8 |  | 5806,00 | 7 | 838 | 0 | 8 |  |
|  | C | 27876 | 5 | 5820 | 0,0 | 5 |  | 0,08 | 5 | 0,103 | 0 | 5 |  |
